# The Armeo Spring as training tool to improve upper limb functionality in multiple sclerosis: a pilot study

**DOI:** 10.1186/1743-0003-8-5

**Published:** 2011-01-24

**Authors:** Domien Gijbels, Ilse Lamers, Lore Kerkhofs, Geert Alders, Els Knippenberg, Peter Feys

**Affiliations:** 1 REVAL Rehabilitation Research Center, Hasselt University, Agoralaan Building A, BE-3590 Diepenbeek, Belgium; 2 BIOMED Biomedical Research Institute, Hasselt University, Agoralaan Building A, BE-3590 Diepenbeek, Belgium; 3 RMSC Rehabilitation & MS Center, Boemerangstraat 2, BE-3900 Overpelt, Belgium

## Abstract

**Background:**

Few research in multiple sclerosis (MS) has focused on physical rehabilitation of upper limb dysfunction, though the latter strongly influences independent performance of activities of daily living. Upper limb rehabilitation technology could hold promise for complementing traditional MS therapy. Consequently, this pilot study aimed to examine the feasibility of an 8-week mechanical-assisted training program for improving upper limb muscle strength and functional capacity in MS patients with evident paresis.

**Methods:**

A case series was applied, with provision of a training program (3×/week, 30 minutes/session), supplementary on the customary maintaining care, by employing a gravity-supporting exoskeleton apparatus (Armeo Spring). Ten high-level disability MS patients (Expanded Disability Status Scale 7.0-8.5) actively performed task-oriented movements in a virtual real-life-like learning environment with the affected upper limb. Tests were administered before and after training, and at 2-month follow-up. Muscle strength was determined through the Motricity Index and Jamar hand-held dynamometer. Functional capacity was assessed using the TEMPA, Action Research Arm Test (ARAT) and 9-Hole Peg Test (9HPT).

**Results:**

Muscle strength did not change significantly. Significant gains were particularly found in functional capacity tests. After training completion, TEMPA scores improved (*p *= 0.02), while a trend towards significance was found for the 9HPT (*p *= 0.05). At follow-up, the TEMPA as well as ARAT showed greater improvement relative to baseline than after the 8-week intervention period (*p *= 0.01, *p *= 0.02 respectively).

**Conclusions:**

The results of present pilot study suggest that upper limb functionality of high-level disability MS patients can be positively influenced by means of a technology-enhanced physical rehabilitation program.

## Background

Multiple sclerosis (MS) is a chronic progressive disease of the central nervous system, mainly affecting young adults, leading to cumulative heterogeneous disability over time. Pharmacological therapies are currently able to slow down the inflammatory-related disability progression, but cannot cure the disease nor restore functionality yet[1]. As such, rehabilitation remains necessary to maximize one's functional status. A vast number of studies has now demonstrated beneficial effects of physical exercise therapy in MS without stating any important deleterious outcome[2].

The physical exercise interventions in these studies were mostly targeting lower limb function and/or ambulatory mobility[2,3]. During the disease course, however, approximately 3 out of 4 MS patients are confronted with upper limb dysfunction,[4] which can manifest bilaterally. As a consequence, a substantial number among them experience a negative impact on important activities of daily living (ADL, e.g. eating or toileting),[5] resulting in dependence and reducing quality of life[6]. Surprisingly, given its relevance, physical rehabilitation studies that specifically target upper limb dysfunction in MS are sparse. By our knowledge, only Mark et al. (2008) have reported, in hemiparetic patients (Expanded Disability Status Scale, EDSS 6.0-7.0; n = 5), significantly improved real-world upper limb use through constraint-induced movement therapy (CIMT)[7]. Obviously, more research is needed to identify the most optimal treatment methodology as well as the treatment potential for different levels of upper limb dysfunction in MS.

In the last decade, computerized robotic and (electro)mechanical devices have been introduced to provide autonomous, high-intensive training for the upper limb[8]. Such devices could hold promise for complementing traditional MS therapy, as therapy time dedicated to arm and hand function training is often limited, principally being indicated in highly disabled MS patients who have a multiplicity of symptoms requiring treatment. On the other hand, training duration and training intensity are known to be key factors for a successful neurological rehabilitation[9]. In particular, this emerging technology enables independent and repetitive movement practice, and this in a motivating, enriched and interactive virtual learning environment in which complex motor tasks, involving central neural pathways related to proprioceptive and visual feedback processing, need to be accomplished. That way, massed exercise according to principles of motor learning,[10] something that is aimed for in rehabilitation,[11] can be established, also by more severely affected individuals who are unable to sufficiently lift their arm against gravity or lacking minimal fine motor capacity to manipulate objects in daily life setting (cf. CIMT).

In stroke, the use of these devices is already well-established. Systematic reviews demonstrated significant improvements in (proximal) upper limb motor strength (Motricity Index, MI) and motor function (Brunnstrom Fugl-Meyer, FM) after robotic/(electro)mechanical-assisted training; however, gains on the ADL level were debatable or modest at best[8,12]. Recently, a study in chronic stroke patients implemented repetitive performance of task-oriented movements in a virtual learning environment through means of the gravity-supporting Therapy Wilmington Robotic Exoskeleton (T-WREX)[13]. Significantly improved patient-rated Motor Activity Log (MAL) scores were stated, representing a better quality and higher amount of affected upper limb use for ADL in the home situation.

In MS literature, so far, robotic/(electro)mechanical technology for the upper limb has barely been engaged as a training tool, certainly not with focus to functional capacity outcome. Two studies have reported the usefulness of end-effector robots as assessment tools for quantifying motor coordination in (a)symptomatic MS patients during the execution of robotic tasks (e.g. reaching tasks towards virtual targets on a screen)[14,15]. Two other studies have investigated the feasibility of an end-effector robot-based rehabilitation protocol for improving upper limb motor coordination, overall reporting, in moderately affected MS patients (EDSS 3.0-6.5; n = 7) who predominantly suffered from ataxia and/or tremor, significant gains in their velocity, linearity and smoothness of reaching movements after 8 training sessions over 2 and 4 weeks respectively[16,17]. This was clinically accompanied with a decrease in ataxia and tremor scores and a significant positive result on time scores of the 9-Hole Peg Test (9HPT). The long-term application of technology for rehabilitating upper limb dysfunction due to paresis has not yet been documented.

Therefore, this pilot study aimed to determine the feasibility of an 8-week mechanical-assisted training program for improving upper limb muscle strength and functional capacity in MS patients with paresis. The training program was given supplementary on customary maintaining care by employing the Armeo Spring (Hocoma AG, Zurich, CH), a gravity-supporting exoskeleton apparatus.

## Methods

### Participants

A convenience sample was recruited among MS patients scheduled at the Rehabilitation & MS Center Overpelt, Belgium. Local neurologists enrolled 10 eligible subjects in present pilot study, which was approved by the appropriate ethical committees. Subjects fulfilled the following inclusion criteria: a definite diagnosis of MS according to the McDonald criteria,[18] and upper limb dysfunction due to evident paresis (characterized by an upper limb MI score ≥ 50 and ≤ 84)[19]. Exclusion criteria were: manifest spasticity (Modified Ashworth Scale > 1)[20] or tremor (Fahn's Tremor Rating Scale > 1)[21] in the upper limb, severe cognitive (Mini-Mental State Examination < 24)[22] or visual (Snellen Test < 50%)[23] deficits interfering with the comprehension or execution of presented virtual reality tasks, or another diagnosis (e.g. orthopaedic) having a major effect on upper limb function. Admitted participants had a high level of general disability and were each wheelchair-bound, as described by an EDSS 7.0-8.5[24]. They all gave written informed consent.

### Apparatus

**Figure 1:**
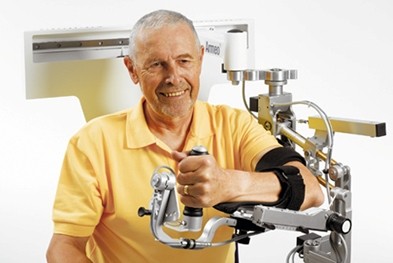
**The Armeo Spring, an exoskeleton apparatus with integrated spring mechanism allowing variable upper limb gravity support**. Photograph courtesy of Hocoma AG.

The Armeo Spring (http://www.hocoma.com/en/products/armeo/armeo-spring/; see also Figure 1), a commercially available replica of the T-WREX,[25] was utilized to train the affected upper limb, being the self-reported dominant side in 8 out of 10 subjects. It is a 5 degree-of-freedom (3 in the shoulder, 1 in the elbow, 1 in the forearm) orthosis without robotic actuators, a so-called passive system. The adjustable mechanical arm allows variable levels of gravity support by means of a spring mechanism. This enables patients, using residual upper limb function, to achieve a larger active range of motion (ROM) within a 3-dimensional workspace than is possible without support[26]. The integration of a pressure-sensitive handgrip additionally allows the execution of graded grasp and release exercises. Through instrumentation of built-in position sensors and software, the Armeo Spring can be engaged as an input device for the accomplishment of meaningful functional tasks (e.g. cleaning a stove top) that are simulated in a virtual learning environment on a computer screen, with the provision of auditory and visual performance feedback during and after practice.

### Experimental design, procedure and training program

An explorative before-after single group research design was applied to examine the feasibility, that is to say the proof of principle, of the training intervention.

An experienced and independent occupational therapist performed the individual setup of the Armeo Spring before training (i.e. establishment of weight compensation, maximal active workspace, and level of exercise difficulty), as well as intermittent supervision under training. The initial amount of gravity support provided by the Armeo Spring was defined based on the subject's ability to maintain the affected arm in a standardized position of 45° shoulder flexion and 90° elbow flexion. Setup features were gradually adjusted at the first training session of each week. If as a consequence increased compensatory movements were observed during task execution, former settings were resumed.

Training frequency was 3 times per week for 8 weeks, or 9 weeks in case the participant missed a training session. One session lasted 30 minutes and consisted of intense repetitive performance of 5 out of 15 virtual reality tasks (5 minutes per task, ranging from gross motor movement when cleaning a stove top, over more precise movement when watering flowers, to subtle strength-dosed movement when picking up an egg), added with 1 patient-preferred therapy game (e.g. car racing or card playing). The mechanical-assisted training was given supplementary on customary care comprising physical and/or occupational therapy aimed at the maintenance of general functional status (e.g. mobilisations to prevent muscle contractures, respiratory exercises, practise of transfers, etc.; 2 to 3×/week, 30 minutes/session).

### Outcome measures

Tests were administered by a single independent researcher, a physiotherapist, before and after 24 training sessions as well as 2 months after training completion.

Upper limb and handgrip muscle strength were determined by means of the MI (normal score = 100) and the Jamar hand-held dynamometer (Biometrics Ltd., Ladysmith, USA). Upper limb functional capacity was assessed with the TEMPA,[27] the Action Research Arm Test (ARAT; normal score = 57)[28] and the 9HPT[29]. For the TEMPA, the median execution time of the 4 unilateral activities (i.e. grasping and moving a jar, pouring water from a jug into a cup, inserting coins in a slot, pinching and moving small objects) was registered. The maximal time limit for each of the 4 TEMPA tasks was 120 seconds, while that of the 9HPT was standardized to 300 seconds. Thus, when a patient was not able to finish a TEMPA task or the 9HPT within the specified time frame, a truncated score of respectively 120 and 300 seconds was given.

Also, after completing the 8-week training program, participants rated their global impression of change in upper limb function compared to the perceived state before the intervention. The utilized 7-point ordinal scale (ranging from 1 = very much improved to 7 = very much worse) was based on the Clinical Global Impression's subscale questioning Change (CGIC)[30].

### Statistical analyses

Normality of the variables was tested applying the Kolmogorov-Smirnov test. Because assumptions of normality were not always fulfilled, and because of the modest sample size, the non-parametric Wilcoxon signed-rank test was implemented to appraise changes in outcome measures after 24 training sessions and at 2-month follow-up relative to baseline. All analyses were done using Statistica (Statsoft Inc., Tulsa, USA). The level of significance was set as *p *< 0.05.

## Results

### Patient compliance and characteristics

One patient dropped out during the study due to personal reasons unrelated to the intervention. This subject was excluded from all analyses. Detailed descriptive characteristics of the participants that completed the training program (*n *= 9) are presented in Table 1. Each of them concluded all 24 training sessions within maximal 9 weeks.

**Table 1:** Patient characteristics (n = 9)

Variable	
**Gender **(m/f)	4/5
**Age **(years)	63 ± 10
**Disease duration **(years)	27 ± 10
**Type of MS **(RR/SP/PP)	0/6/3
**EDSS**	7.9 ± 0.5
**Trained upper limb **(D/ND)*	6/3

Values are mean ± standard deviation, or number.

RR, relapsing remitting; SP, secondary progressive; PP, primary progressive; EDSS, Expanded Disability Status Scale; D, dominant; ND, non-dominant.

* 2 out of 3 non-dominant limbs have become dominant limbs over time because of paralysis of the initial dominant limb.

### Effects of the Armeo Spring training program on upper limb muscle strength and functional capacity

Baseline values of the outcome measures and changes over time are provided in Table 2. Armeo Spring training yielded no significant alteration in upper limb muscle strength, although the mean MI score improved subsequent to the intervention, sustaining gain at follow-up. Hand grip force measured with the Jamar remained stable throughout the whole study.

**Table 2:** Changes in outcome measures with Armeo Spring training (n = 9)

Variable	Baseline value	Δ after 24 training sessions	p of Δ after 24 training sessions	Δ at 2-month follow-up, relative to baseline	p of Δ at 2-month follow-up, relative to baseline
**MI**	72 ± 8	4 ± 7	0.07	6 ± 9	0.08
**Jamar **(kg)	14,3 ± 9,1	0,2 ± 4,5	0.51	0,0 ± 5,7	0.67
**TEMPA **(s)	56,4 ± 44,1	-23,6 ± 27,4	0.02*	-26,8 ± 27,0	0.01*
**ARAT**	45 ± 13	4 ± 11	0.31	5 ± 7	0.02*
**9HPT **(s)	157,1 ± 114,6	-47,8 ± 59,4	0.05+	-47,0 ± 76,9	0.09

Values or mean ± standard deviation.

Δ stands for change in outcome measures; *p < 0.05; ^*+ *^trend towards significance.

MI, Motricity Index; ARAT, Action Research Arm Test; 9HPT, 9-Hole Peg Test.

**Figure 2:**
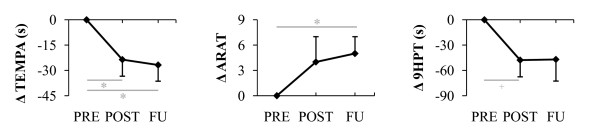
**Effects of Armeo Spring training on upper limb functional capacity parameters**. Changes in outcome measures (*Δ*) were measured after 8 weeks of training (POST) and at 2-month follow-up (FU), relative to baseline (PRE). Vertical bars show 1 standard error; *p < 0.05; ^+ ^trend towards significance. ARAT, Action Research Arm Test; 9HPT, 9-Hole Peg Test.

**Figure 3:**
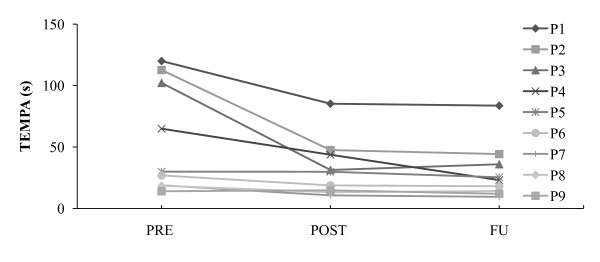
**Case profiles of time performance on the TEMPA**. Outcomes were measured at baseline (PRE), after 8 weeks of Armeo Spring training (POST), and at 2-month follow-up (FU). P, patient.

Significant improvements were particularly found in functional capacity parameters (see Figure 2). At completion of the training program, the functional activities of the TEMPA were performed significantly faster compared to baseline, while time scores on the 9HPT gave evidence of a positive trend. ARAT scores increased 4 points on average, not being significant however. Largest gains were observed in subjects most affected at baseline, more specifically in 4 individuals who initially required a TEMPA execution time of more than 60 seconds (see Figure 3 in illustration of this finding) and a 9HPT execution time of more than 180 seconds, besides scoring less than 41 points on the ARAT. In fact, these 4 subjects were not able to accomplish one or more TEMPA tasks (all 4 individuals) or the 9HPT (2 out of 4 individuals) within the specified maximal time frame before the intervention, while most of them were capable after the intervention (3 out of 4, and 4 out 4 individuals respectively). At 2-month follow-up, results on the TEMPA and ARAT revealed even greater and for both measures significant gains relative to baseline than immediately after the intervention period, despite the fact that in the meantime no supplementary mechanical-assisted training had taken place. The 9HPT outcomes approximated the post-training performance levels.

After finishing the training program, 3 participants rated themselves much improved, 2 participants rated themselves moderately improved, and 4 participants noted no change on the CGIC, without stating any side effects. Interestingly, the 4 subjects who showed greatest responsiveness on the functional capacity parameters were among those declaring much (3 individuals) and moderate (1 individual) self-perceived improvement.

## Discussion

This pilot study reports on an 8-week technology-enhanced training program for improving upper limb muscle strength and functional capacity in MS patients with paresis. The gravity-supporting Armeo Spring was employed as a training tool assisting participants to additionally and independently practice task-oriented movements in a virtual real-life-like learning environment. Importantly, significant gains in the functional capacity outcome measures were found after completion of the intervention period, which sustained or even progressed at 2-month follow-up.

In MS, limited literature is available on rehabilitation of upper limb dysfunction, neither with regard to traditional physical therapy in general, nor with regard to technology-enhanced physical therapy in particular. Previously, a 2- and 4-week robot-based rehabilitation protocol, applied in moderately affected patients (EDSS 3.5-6.0) who predominantly suffered from cerebellar symptoms like ataxia and/or tremor, led to improved upper limb motor coordination as measured through robotic parameters, ataxia and tremor indices, and the 9HPT[16,17]. Current investigation implemented mechanical-assisted training over a longer period of 8 weeks as a treatment modality supplementary on customary maintaining care. Beneficial effects were noted, particularly on the functional capacity level, and this mainly in subjects whose upper limb function was most affected at baseline (i.e. initially having a TEMPA execution time > 60 seconds, 9HPT execution time > 180 seconds, ARAT score < 41 points). It were also these individuals that, examined by the CGIC after finishing the training program, perceived at least moderate improvements in their upper limb function compared to the status before the intervention. Patient's quotations were: 'Combing my hair goes easier', 'I can scratch my nose again when it itches', or 'I'm better able to hold a book and turn pages'. Given that precarious arm and hand dysfunction often occurs in a later stage of MS, it is noteworthy that the above findings were obtained in high-level disability patients with an EDDS ≥ 7, a patient subgroup that as far as we know has not been studied before in the context of (technology-enhanced) physical rehabilitation[2]. Our study results suggest that the upper limb of such persons, who are already wheelchair-bound, is still trainable with profits being established in a functionally relevant way.

It is acknowledged that MS and stroke can present themselves with different clinical symptoms. Nonetheless, it is supportive to notice that the reported effects of Armeo Spring training in MS are in concordance with the outcomes of a recent randomized clinical trial (RCT) in stroke patients with chronic hemiparesis (cf. two distinct pathologies showing similar upper limb dysfunction caused by upper motor neuron lesions)[13]. This RCT also demonstrated, subsequent to 8 weeks of gravity-supported T-WREX training, functionally relevant changes in the use of the affected upper limb in terms of significantly improved patient-rated MAL scores, besides significant gains in active reaching ROM and the FM. In both studies in MS and stroke, handgrip force measured with the Jamar showed no significant alteration. This might be because especially proximal muscles around the shoulder girdle, shoulder and elbow joint were exercised during the execution of virtual reality tasks. The pressure-sensitive handle integrated in the exoskeleton systems effectively allows grasp and release exercises, but these only need to be performed submaximally in part of the tasks. In present research, the MI measuring overall upper limb muscle strength improved, albeit non-significant. A less pronounced gain in strength is not entirely surprising given that the Armeo Spring(/T-WREX) device provides anti-gravity support, notwithstanding the fact that this support had (slightly) decreased in all subjects at the end of the training period.

Movement practice in a virtual environment with the Armeo Spring may rather be considered as dexterity training, by which (partial) relief of the upper limb's weight enables the more severely affected patient to actively produce a larger ROM within a 3-dimensional workspace[31]. Dexterity is hereby defined as the ability to address spatial and temporal accuracy necessary to make the movement meet environmental demands[32]. So mechanical-assisted therapy in a virtual workspace engages not just repeated *use *of the upper limb, but involves repetitive and active exertion of goal-directed movements, with enlarged ROM and superior multi-joint coordination, during the practice of complex motor tasks in an enriched *learning *environment. Focus on dexterity during (technology-enhanced) task-oriented training is deliberately wanted by therapists,[33] and could have been a main driver for the improved functional outcome of the upper limb in both MS and chronic stroke patients[11].

The improved functional capacity is of importance as systematic reviews assessing the effectiveness of robotic/(electro)mechanical-assisted training in stroke mainly demonstrated significant gains in upper limb motor function, contrary to benefits on the ADL level which were less pronounced[8,12]. In the selected studies for review, emphasis was rather put on 2-dimensional goal-directed instead of 3-dimensional task-oriented training, which might have contributed to the lack of effectiveness for functional recovery. However, the limited contrast between experimental and control interventions can be another issue in this regard. In the recent RCT of Housman et al. (2009), patients receiving control therapy in the form of conventional table top exercises, positively exhibited similar improvements on the outcome measures as patients receiving mechanical-assisted training with the T-WREX, except for a modest sustained gain on the FM at 6-month follow-up in favour of T-WREX, while participants expressed their preference for T-WREX training after a single-session crossover treatment[13]. It seems unlikely that robotic/(electro)mechanical-assisted training will arrive at better results than another training modality/therapist-mediated training under the premise that the content, frequency, amount and intensity of therapy are comparable[34]. Yet, rehabilitation technology enables stimulating as well as cost-effective practice, since it can be performed on a relatively autonomous and additional basis, also by a more disabled patient population as the one in the present study that does not necessarily meet the selection criteria for a functional training modality such as CIMT[35].

Another important finding in current investigation is the fact that the noted effects on the functional capacity level sustained or even progressed at 2-month follow-up. Analogue statements were made in the above mentioned T-WREX study in stroke, where functionally relevant changes revealed by the MAL showed greater significant improvement at 6-month follow-up relative to baseline than after the 8-week intervention period. This patient-reported index supports our assumption that beneficial effects of technology-enhanced training plausibly culminated an increased spontaneous use of MS patients' paretic upper limb in the habitual life situation, retaining or further enhancing outcome over time. It also suggests that 8 weeks of repetitive weight-supported practice in a virtual setting can work out transferred and durable benefits in non-weight-supported real-world upper limb functionality in either chronically affected MS and stroke patients. Within this context, it is regretful that the two studies in diverse pathologies applied other outcome measures on the various domains of the International Classification of Functioning, Disability and Health (ICF),[36] hindering direct comparison of the extent of improvement between neurological conditions and possible differential effects of different total training times in both investigations. Future research in MS should therefore consider the inclusion of parameters that are frequently used in stroke, such as the MAL (although not yet fairly applicable in MS as it compares the affected with the non-affected upper limb, whereas motor symptoms can manifest bilaterally in MS patients) and the FM index[37,38].

Present study is not without limitations, while the underlying mechanisms for changes in motor performance are not fully clear. Firstly, this pilot investigation applied a before-after single group research design in a limited sample size without incorporation of a control group, given that the aim of the study was to ascertain proof of principle and treatment potential of mechanical-assisted upper limb training in MS patients with paresis. Nevertheless, it is believed that the reported changes in upper limb functionality reflect true improvement rather than a practice effect related to repeated test execution, since one would not expect to perceive substantial gains in chronically and severely disabled MS patients (EDSS ≥ 7)[39]. Besides, the participants were familiar with the outcome measures as these are part of the routine clinical assessment administered at the Rehabilitation and MS Center Overpelt. Secondly, in retrospect, implementation of a parameter on the ICF's participation level examining upper limb use in the daily life, such as the subjective MAL or an objective wrist actigraph like proposed by Kos et al. (2007),[40] would have made this research more solid. Those instruments are closer to demonstrate the ultimate rehabilitation objective, which is having a positive impact on the community function of patients. Also, the included functional capacity outcome measures do not allow explanation about the underlying mechanisms on the basis of improved motor performance. Neural plasticity has already been shown in MS, conceivably moderating the clinical manifestations of the disease[41]. Given that the applied practice modality in present investigation implemented adaptive motor learning,[42] one could question oneself if this may have led to the stimulation of restorative brain plasticity resulting in genuine upper limb motor recovery. On the other hand, the functional gain could also be owing to the usage of more efficient compensation strategies (e.g. enhanced trunk and proximal arm movement) or, very realistically, the overcoming of learned non-use secondary to MS. Future research should regard the application of both kinematical (e.g. accelerometry) and neurophysiological (e.g. transcranial magnetic stimulation) measurements to determine quality of movement and to comprehend the neural substrates underlying motor performance.

## Conclusions

This pilot study is the first one to provide indications that technology-enhanced physical rehabilitation is effective for improving upper limb functionality in high-level disability MS patients with paresis, and this in a durable manner. Beneficial effects were mainly noted in individuals most affected at baseline. Further RCTs including a broader assessment are warranted to confirm and elaborate these results.

## Competing interests

The authors declare that they have no competing interests.

## Consent

Written informed consent was obtained for publication of the accompanying image. A copy of the written consent is available for review by the Editor-in-Chief of this journal.

## Authors' contributions

DG and PF conceived of the study, participated in its design and coordination, and drafted the manuscript. IL, GA and EK co-operated in the study design and performed data collection. DG and PF carried out the statistical analysis. LK provided project management and consultation. All authors read and approved the final manuscript.
